# The Value of Sanatoriums under the Insurance Bill

**Published:** 1911-10-07

**Authors:** 


### SANATORIA: THEIR USE AND ABUSE.

Contributions, experiences and questions relating to Sanatoria, their Administration and Work are invited.

### The Value of Sanatoriums under the Insurance Bill.

At the time Mr. Lloyd George introduced his Insurance Bill he stated that it was high time we got rid of tuberculosis in this country, and for this purpose he proposed to allocate a certain sum of money to the building of sanatoria. To those who are thoroughly conversant with the problem of pulmonary tuberculosis, it must be quite obvious that sanatoria alone are not going to wipe out this disease. It is a social as well as a medical problem, and in this connection the old adage that "Prevention is better than cure" is particularly applicable to this disease. The sanatorium is an absolutely necessary link in the chain of prevention and abolition of tuberculosis, but it should be thoroughly realised that it is only a link, and preventive measures must be vigorously adopted to stop the supply of material to such institutions. One can only suppose the Chancellor of the Exchequer used the word "sanatorium" as meaning any building which was devoted to the prevention and treatment of tuberculosis, and not meaning a sanatorium in the sense used by workers in tuberculosis. The term "sanatorium," therefore, should, to be effective, include an up-to-date sanatorium in the country, hospitals, or wards attached to hospitals, and infirmaries for tuberculous patients who are not well enough to go to the sanatorium, or who fail after treatment there, and who will never be well enough to earn their living. There should be homes for the dying. A laboratory for clinical and research work must also be included in the term.

### The Limitations of Sanatoria.

The sanatoria of the past have not come up to the expectations of those people who, ten years or so ago, stated that if enough of such institutions were built tuberculosis would be a thing of the past; and one has not far to seek for the reasons why they have partly failed in their object. In the first place, no allowance was made for failures. Secondly, many small institutions were built, and, in consequence of their size, were not able to offer adequate salaries for the medical officers, the natural result being that those medical men who had suffered from tuberculosis were often enough glad, for their own sakes, to accept the posts. It does not follow, because such a man has had consumption, that therefore he will make a successful superintendent of a sanatorium. Thirdly, it was not realised that something more than fresh air and good feeding is necessary to restore to health all cases of tuberculosis; all persons who have become infected by the tubercle bacillus can be placed into one of the four following classes: (1) Those who recover without being in any way aware of the infection; (2) those who recover by going abroad, or to the seaside, rest and change of air having been sufficient to restore their resistance; (3) those who recover when placed under ideal conditions in a sanatorium; (4) those who would not recover under any circumstances.

The number of those infected by the bacillus who are in Class 1 we cannot estimate. Those who go into Class 2 are those people who have recovered under the influence of the so-called open-air treatment, or by a residence in Switzerland or at the seaside. Class 3 are those patients who have been failures too often in the past, because nothing has been done to raise their specific resistance to tuberculosis. Class 4 are the failures about whom I have already spoken, and they form a very definite class.

### Aims of Modern Sanatoria.

Modern sanatorium treatment aims much higher than merely putting the people in fresh air and giving them plenty to eat. In addition to such treatment, a definite effort is made to raise the patient's resistance to tuberculosis, either by properly controlled auto-inoculation or by tuberculin treatment. By using these extra means a very much larger number of patients will be turned out in hard condition, not over fat, and fit for work. Even with this treatment the sanatorium is not an infallible specific; there always will be people whose resistance cannot be raised, and who, in spite of everything, cannot be restored to their full working capacity. The sanatorium, too, will suffer, in addition, from the fact that quite a considerable number of people succumb to the infection from lack of sufficient food owing to a scarcity of work. If these patients are admitted to the sanatorium, although they may be completely restored to their full working capacity by treatment, when they return to the same conditions under which they contracted tuberculosis, they will, in all probability, break down once more. This is not the fault of the sanatorium, but it is a side of the work which must be provided for in any comprehensive treatment for tuberculosis.

There is a feeling that the building of new country sanatoria is useless, but the treatment on the lines indicated above is the very best treatment that we know of at the present time, and we should use the best we have until something better is obtainable. Supposing only 30 per cent. of the patients in sanatoria remain well for five years after discharge. Should we deny treatment because of such a low percentage of permanent results, and simply allow these people to die? It is our duty to see that these patients are treated, for three reasons: First, it would do all that is humanly possible to insure them against the risk of developing into hopeless consumptives; secondly, because it would prevent their spreading infection to others; thirdly, it would give them a practical education in sound hygienic principles.

